# The digital transformation of clinical teaching: a review of AI and data-driven quality assurance systems in medical education

**DOI:** 10.3389/fpubh.2026.1915158

**Published:** 2026-08-17

**Authors:** Kexin Zhao, Ningning Wang, Zhicai Xie

**Affiliations:** 1 Technology and Information Department, The Second People's Hospital of Longgang District, Shenzhen, Guangdong Province, China; 2 The Second People's Hospital of Longgang District, Shenzhen, Guangdong Province, China; 3 Beijing University of Chinese Medicine Shenzhen Hospital (Longgang), Shenzhen, Guangdong Province, China

**Keywords:** artificial intelligence, clinical teaching, digital health education, learning analytics, medical education, quality assurance, simulation

## Abstract

**Background:**

The use of artificial intelligence (AI) and other data-driven technologies is changing how medical education and clinical teaching are conducted. The traditional model of learning will not allow us to keep up with the sheer amount of new knowledge being produced, the move to competency-based education, or the need for learning that can be scaled and tailored to fit individuals. To examine how AI is being used in clinical education and evaluate its impact on teaching, learning, assessment, and quality assurance.

**Methods:**

A review of the literature was conducted to examine the applications of AI in clinical education, including adaptive learning, intelligent tutoring, virtual patients, simulation, automated assessment, and data-driven quality assurance.

**Results:**

AI has been shown to enhance the engagement of learners, create flexible learning opportunities, provide timely feedback for information on a learner's progress, and provide ongoing objective assessment of clinical competency. The use of learning analytics and predictive modeling enables institutions to monitor the progression of the learner, identify learners at risk, optimize the design of their curriculum, and make evidence-based academic decisions. Future innovations in medical training environments could be possible through the use of digital twins, immersive simulation, and explainable AI. However, there remain significant challenges to the widespread implementation of these technologies, such as bias in algorithms, lack of transparency, issues related to data privacy, unregulated use, and the limitations of current systems. Research to date has demonstrated great variation across studies and has not provided longitudinal data to support any conclusions about the long-term impact of using AI tools.

**Conclusions:**

Despite the great advantages offered by using AI-based technologies in clinical education, Successful integration will require robust governance frameworks, faculty development, equitable access, rigorous validation, and continuous evaluation to ensure ethical, effective, and sustainable implementation.

## Introduction

A significant change to medical education is taking place as rapid advances in digital technology, especially artificial intelligence (AI) and big data analytics, continue to drive this transition (1). Traditional clinical education models that rely heavily on observational learning and subjective evaluation will be increasingly challenged by the vast amounts of medical knowledge that are expanding exponentially, the growing complexity of patients, and the global transition toward competency-based education. The current estimate is that the volume of medical knowledge is doubling every few months, making it difficult for traditional medical school curricula to be able to keep up to date and fully knowledgeable (2). As such, there is an urgent requirement for scalable, adaptable, and data-informed educational frameworks. To meet these challenges, AI technologies, including machine learning, natural language processing, and generative AI, are increasingly being incorporated into the medical education system. New research indicates a significant number of medical schools have adopted some form of AI-enabled technology: intelligent tutoring systems, virtual patients, and automated assessment systems, among others (3). These technologies create personalized learning pathways, provide real-time feedback to students, and support objective assessments of clinical competence, resulting in a supportive and interactive learning environment that will improve students' overall academic success. In addition, simulation technologies and immersive learning tools are continuing to advance toward providing safe, repeatable, and standard clinical training experiences (4). Additionally, as educational effectiveness continues to evolve through data-driven quality assurance models, learning analytics and predictive modeling will continue to allow schools to consistently track the success of their students, identify high-risk students at an early stage, and implement evidence-based practices to improve the curriculum (5). The use of digital dashboards and automated feedback will also provide institutions with an additional level of transparency into their educational outcomes at both the institutional and regulatory levels (6). Although the number of medical schools taking advantage of AI has grown significantly since 2015, challenges related to data governance and ethics continue to inhibit the pace and the quality of successful implementation of AI technology. This study seeks to address this gap in the literature by providing an overall synthesis of AI clinical applications across three domains: teaching, assessment, and quality assurance, based on data, as well as identifying barriers and providing guidance for equitable, sustainable, student-centered digital change within the clinical education system.

## Methodology

**Figure 1:**
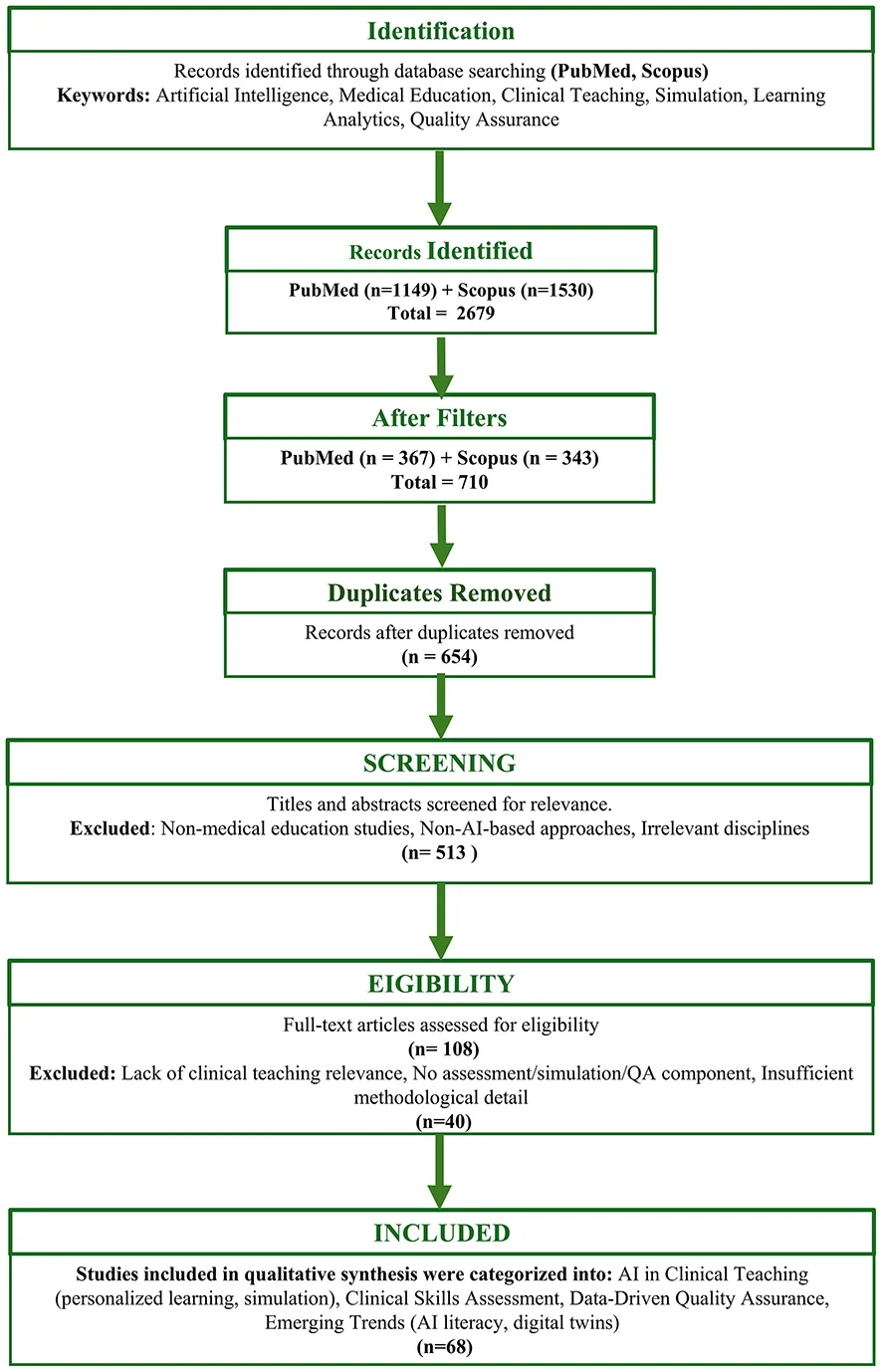
PRISMA-informed flow diagram illustrating the study selection process for literature on artificial intelligence and data-driven quality assurance systems in medical education.

This study is a structured narrative review that employed a systematic literature search and a PRISMA-guided study selection process to enhance transparency and methodological rigor. The retrieved evidence was synthesized narratively and organized into thematic categories rather than subjected to quantitative meta-analysis. We conducted a structured and comprehensive approach to the literature published concerning AI and Data-Driven Quality Assurance in Medical Education to synthesize pertinent evidence on this topic (7). A systematic search of the relevant literature was performed using the primary electronic databases PubMed and Scopus for publications indexed from 2015 through 2026. PubMed and Scopus were utilized due to their wide-ranging coverage of biomedical, clinical, and interdisciplinary literature pertinent to research on artificial intelligence and medical education. The literature search was conducted in January 2026. The search included terms such as “Artificial Intelligence,” “Medical Education,” “Clinical Education,” “Learning Analytics,” “Simulation,” and “Quality Assurance” (8). The detailed search strategy is provided in Supplementary material S1. After searching our databases, we located 2,679 relevant records. The records were filtered, and duplicates were removed, leaving 654 records for screening. Titles or abstracts were screened, resulting in a sample of 108 articles that went through a full-text eligibility process; of those, 68 studies were included in the qualitative synthesis (Figure 1). Original research articles as well as review articles (i.e., systematic and narrative reviews) were considered to provide the most inclusive representation of the subject matter being analyzed. The only studies included in this review were those that specifically discussed how AI has been implemented in Clinical Education (Teaching) and in the Assessment of Clinical Competency, along with Simulation (9). Studies that were not directly related to the field of Medical Education and/or that did not involve an AI or Data-Driven approach to improve the Quality Assurance of Medical Education were excluded (10). A PRISMA guide was used during the literature search for topic relevance, selection, and screening to provide transparency and methodological rigor (11). Study selection was performed by two independent reviewers, and any disagreements were resolved through discussion and consensus. Selected studies were critically evaluated and organized by theme so that a coherent synthesis of current advancements, challenges, and future directions in clinical education through digital transformation is provided. The studies that were reviewed were organized into four domains of interest: AI in Clinical Education, Assessment, Simulation and Data-Based Quality Improvement. The collected evidence was synthesized in a descriptive style and classified into categories to provide a clear understanding of the applications, challenges, and prospects of using AI and data-driven quality assurance systems in medical education. Because this study was conducted as a structured narrative review rather than a formal systematic review, a formal methodological quality or risk-of-bias assessment of individual studies was not performed.

## AI in clinical teaching

### Personalized learning and adaptive education

Clinical education may also undergo a huge transformation through AI-powered platforms, where adaptive and personalized educational platforms will utilize new machine learning algorithms that can analyze clinical student performance, behavior, and knowledge gaps to develop customized clinical student learning experiences (12). In comparison to a static curriculum, which provides prescriptive delivery methods, adaptive environments change and adapt in real time based on a student's interaction with the content and results, resulting in better retention of information and increased motivation to learn (13). AI-based Intelligent Tutoring Systems (ITS) also provide continuous non-summative assessment and instant/permanent feedback that allow students to make quick corrections to their mistakes, supporting a competency-based medical education approach in which progression is based on the demonstration of competency rather than time (14). By utilizing a variety of different types of multimodal data, i.e., assessment scores, engagement data, AI provides a comprehensive picture of student progress and development. The use of predictive analytics also allows educators to identify those students who are at risk of failure early in the process, thus allowing them to implement timely interventions (15). It is important to note that the push toward personalization and competency-based advancement is not exclusive to AI-powered tools. Data-informed evaluation frameworks are prerequisites for effective adaptive systems. For instance, in contexts like general practitioner training—where experienced specialists switch to primary care—comprehensive assessment frameworks like the Kirkpatrick model (Reaction, Learning, Behavior, Result) are employed to evaluate training effectiveness. Studies analyzing factors influencing the outcomes of such transition programs, such as trainee background, training institution resources, and curriculum alignment with primary care competencies, provide the essential performance data and understanding of learner variability upon which future AI-driven personalization could be built. Overall, AI-based personalized learning represents a dramatic change to a more efficient and learner-centered educational model (Table 1).

**Table 1:** Applications of AI in personalized learning in medical education.

Sr. no.	AI Tool/technology	Key function	Application in medical education	Educational outcomes	References
**1**	Intelligent tutoring systems (ITS)	Provide adaptive instruction based on learner responses	Personalized teaching of clinical concepts and case-based learning	Improved knowledge retention, individualized learning pace	(65)
**2**	Adaptive learning platforms	Adjust content difficulty and sequence using performance analytics	Customized curriculum delivery in preclinical and clinical subjects	Enhanced learning efficiency and competency attainment	(66)
**3**	Machine learning algorithms	Analyse learner data to identify patterns and predict performance	Identification of knowledge gaps and prediction of at-risk students	Early intervention improved academic performance	(67)
**4**	Natural language processing (NLP)	Interpret and evaluate textual and verbal inputs	Assessment of clinical reasoning, documentation, and communication skills	Enhanced feedback quality and communication proficiency	(68)
**5**	Learning analytics systems	Track and visualize student engagement and performance metrics	Monitoring learner progress through dashboards and reports	Data-driven decision-making and continuous performance improvement	(69)
**6**	Recommender systems	Suggest relevant learning resources based on learner needs	Personalized content delivery (e.g., articles, videos, case studies)	Increased engagement and targeted knowledge acquisition	(70)
**7**	Chatbots and virtual assistants	Provide real-time academic support and query resolution	24/7 assistance for medical students in understanding concepts	Improved accessibility to learning resources and learner satisfaction	(71)
**8**	Predictive analytics tools	Forecast future performance using historical and real-time data	Identifying students requiring additional support or remediation	Reduced dropout rates and improved learning outcomes	(5)
**9**	AI-based assessment systems	Deliver automated, continuous formative assessment	Real-time quizzes and competency-based evaluations	Objective assessment and timely feedback	(72)

### AI-enhanced simulation and virtual patients

AI has rapidly changed the way simulation-based education is implemented in healthcare, including the development of AI-based intelligent virtual patients and immersive experiential learning platforms (16). AI-based simulation technologies give students the opportunity to practice their clinical thinking, communication, and decision-making skills using realistic simulation-based patients and to practice them in a risk-free manner. Advanced simulation technology (Natural language processing, Generative AI) will facilitate students' interactions with simulated patients that are approaching the real-life experience of how these patients may present to a healthcare provider, including variability (17). As simulation becomes more widely accepted as an effective method of teaching and learning, learners will gain experience through repetition, receive feedback on their performance, and subsequently improve their skills over time. The research supports that simulation-based training develops clinical competency, confidence, and readiness for real-world clinical experiences (18). For example, many AI-based virtual patient simulations, including AI-based intelligent clinical simulations and chatbots powered by large language models, are being used to simulate complex patient interactions in a clinical simulation environment (19). These systems allow learners to practice history-taking, diagnosis, and communication skills in a controlled and standardized environment, thereby improving clinical preparedness and decision-making abilities. In addition to increasing the accessibility and availability of simulated patients, AI is facilitating the standardization of education across institutions and diminishing the variability associated with clinical exposure. In addition, technology provides solutions for challenges associated with limited availability of patients, ethical concerns around patient encounters, and the variability in the complexity of clinical cases experienced during a student's clinical rotation (20). Using technology (e.g., virtual reality, augmented reality) in conjunction with simulations provides an immersive experience for students and allows for broad exposure to a variety of different types of clinical experiences.

### AI in clinical skills assessment

Traditionally, evaluation of clinical competence is subjective, creating variability and bias across assessors in a way that undermines the integrity of clinical assessments. AI is being utilized to create easier means of evaluating clinical skills in a more objective, standardized, and data-driven way than has previously been possible through traditional methods. By using machine learning algorithms, AI can analyse many dimensions of a clinician's performance, including procedural accuracy, time efficiency, decision-making behavior, and effectiveness of communication (Table 2) (21). AI-based platforms are capable of rapidly analyzing vast amounts of data contained within electronic records, simulations and other digital inputs to produce a comprehensive performance profile for each clinician. As a result, clinicians can be distinguished from one another across the spectrum of expertise in an accurate and reliable manner, allowing for greater consistency and quality between the evaluations completed by different assessors (22). In addition, by taking advantage of new technologies such as computer vision and sensors, real-time evaluations of procedural skills can be completed. Natural language processing (NLP) can also facilitate the assessment of clinical communication. Despite the advantages, data bias, lack of transparency, and resistance to adoption help to restrict the capabilities that AI has to enhance clinical evaluation through increased precision, scalability, and objectivity (23). There are many ways in which current evidence is very limited with respect to AI clinical assessment. Most of these studies examine clinical assessment with AI within a controlled environment or simulated environment, meaning they may not be reflective of a true clinical environment where actual clinical assessments will take place (24). Furthermore, due to the variability of the different studies in terms of study design, sample size, and evaluation metrics, it is difficult to compare outcome measures across the different AI systems. Longitudinal studies investigating the effects of AI-based evaluations over extended time frames on both clinical proficiency and patient outcomes are limited. Additionally, without standard verification and benchmarking protocols, establishing the consistency and reproducibility of AI technology may be challenging. Future efforts must consist of multi-level projects that involve large-scale research to generate accurate analyses of AI's use in the clinical setting by employing standardized methods of evaluation (25).

**Table 2:** AI applications in clinical skills assessment and their outcomes.

Sr. no.	Assessment type	AI method	Evaluation focus	Outcome	References
**1**	Procedural skills	Computer vision	Precision, motion tracking	Objective skill measurement	(73)
**2**	Clinical decision-making	Machine learning	Diagnostic accuracy	Improved clinical reasoning	(74)
**3**	Communication skills	NLP, speech analysis	Clarity, empathy	Standardized communication evaluation	(75)
**4**	OSCE assessment	Automated scoring systems	Checklist adherence	Reduced examiner bias	(75)
**5**	Simulation-based assessment	AI simulation analytics	Response accuracy	Real-time feedback and preparedness	(4)
**6**	Surgical training	Deep learning + vision systems	Error detection, efficiency	Enhanced surgical precision	(76)
**7**	Documentation skills	NLP	Completeness, terminology	Improved clinical documentation quality	(68)

## Data-driven quality assurance in medical education

### Learning analytics and performance monitoring

**Figure 2:**
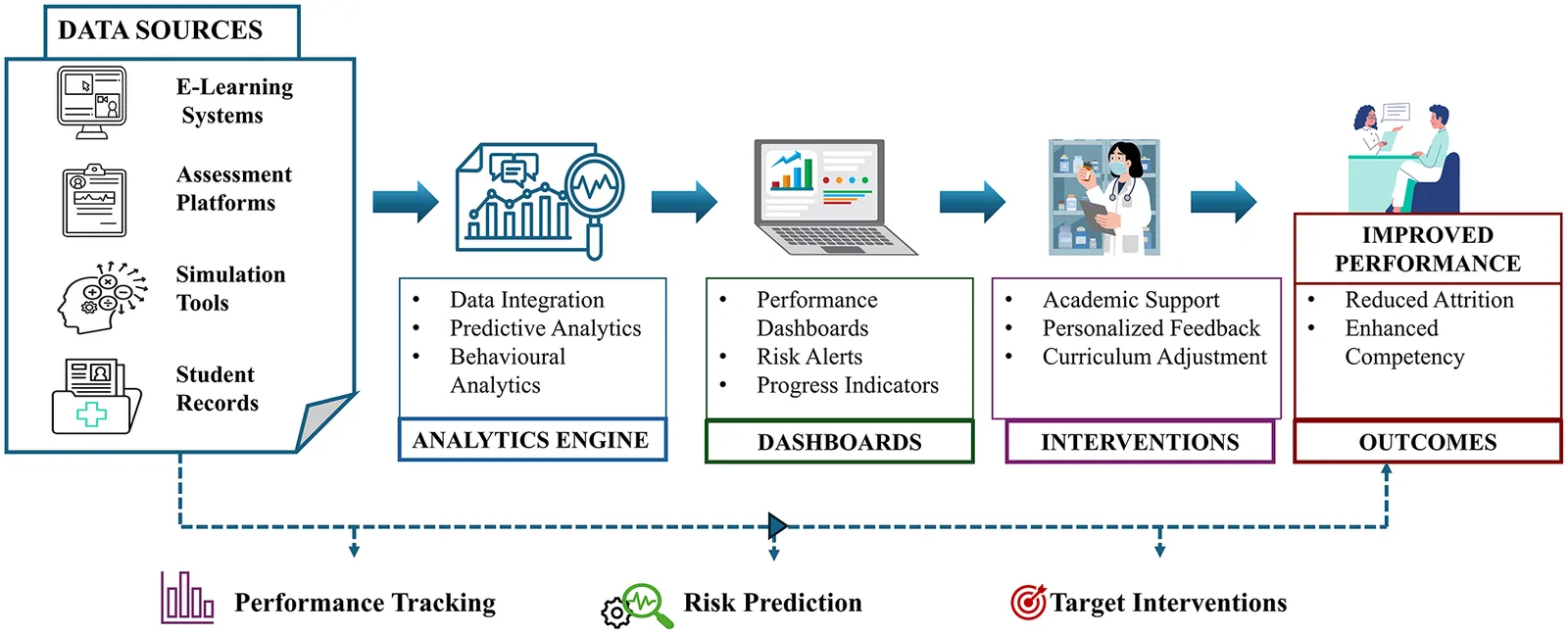
Framework of learning analytics in medical education illustrating the workflow from multiple data sources (e-learning systems, assessment platforms, simulation tools, and student records) to analytics processing, dashboard visualization, targeted academic interventions, and improved educational outcomes.

The use of data-driven systems in medical education has made it possible to continuously and objectively track the performance of learners and the impact of the institution on learners. Learning analytics involves collecting, processing, and analyzing large amounts of educational data from digital learning platforms, simulation systems, and various assessment tools (Figure 2) (26). Within this data are records of learner engagement, testing results, time spent on a task, and behavioral interactions, which give educators and administrators a full picture of how each learner is progressing. A concrete application of this principle in addressing systemic workforce challenges can be seen in the evaluation of large-scale transition training programs. In China, for example, the “General Practitioner Training” program is a key national strategy to rapidly expand the primary care workforce. Researchers have applied learning analytics methodologies to evaluate this program's effectiveness within the specific context of tiered healthcare delivery systems. By integrating trainee final assessment scores (learning layer) with multi-source survey data from trainees, colleagues, and patients (reaction, behavior, and result layers), studies aim to construct localized evaluation frameworks and identify key factors—such as training institution quality, curriculum design, and trainee motivation—that influence the development of primary care competence. This approach exemplifies how data-driven quality assurance is used not only for traditional students but also for the continuing professional development of practicing physicians, directly supporting health policy goals like tiered healthcare delivery and strengthening primary care services. Using predictive analytics allows institutions to identify students at risk early enough to provide appropriate academic intervention or a learning remediation strategy in a timely fashion (27). Continuous performance tracking supports competency-based education by longitudinally assessing clinical skills and knowledge acquisition. Predictive analytics models are also increasingly being used to predict how well a student will perform academically, to allocate funds appropriately, and to aid in making strategic academic administration decisions (28). The result of utilizing a data-driven approach is improved educational efficiency and effectiveness, reduced attrition, and, ultimately, improved student success. Many institutions are deploying dashboard-type learning analytics systems to give immediate visual feedback to educators about student engagement, performance, and progression. This type of dashboard will allow an educator to identify “at-risk” learners early on so that they can put in place targeted interventions, enhancing the academic performance of both the individual learner and the learner's institution (29).

### Curriculum optimization and feedback systems

AI tools have become an important component of optimizing the curricula of medical schools by helping identify inefficiencies, redundancy, and gaps in the delivery of educational material; through analyzing the student performance data and learner feedback (to adjust curriculum dynamically) in a manner that is aligned to competency-based standards (Table 3). As well as AI's role in assisting with the refinement of curricular content, AI provides tools for curriculum mapping to assist the educator in ensuring better alignment between learning objectives, teaching activities, and assessment strategies (30). AI-driven automated grade feedback systems provide educators with a greater degree of transparency and responsiveness. They produce real-time, data-driven information about students' performance and will ultimately result in continuous improvement of the process of teaching and learning (31). For example, by utilizing trends from assessment data to identify and address common misconceptions among learners, therefore directing instructional support where it is most needed. The major areas AI tools can be utilized include curriculum mapping, automated assessment production, and creating feedback loops in order to continuously improve the quality of medical education. To provide Adaptive, evidence-based, and Responsive solutions to the evolving educational needs of the healthcare sector (20).

**Table 3:** AI applications in curriculum optimization and quality improvement.

Sr. no.	Curriculum function	AI application	How it works	Impact on curriculum design	Educational outcome	References
**1**	Curriculum gap analysis	Learning analytics	Analyses student performance across modules to detect weak areas	Identification of missing or underperforming topics	Improved content coverage and knowledge balance	(77)
**2**	Content alignment	AI-based curriculum mapping	Aligns learning objectives with teaching and assessment data	Ensures coherence between objectives, teaching, and evaluation	Better competency-based education implementation	(78)
**3**	Curriculum sequencing	Machine learning models	Determines optimal order of topics based on learner progression data	Logical structuring of course flow	Enhanced learning efficiency and retention	(79)
**4**	Assessment-curriculum linkage	Automated assessment analysis	Evaluates assessment results to refine teaching content	Adjusts curriculum based on student performance trends	Improved alignment between teaching and evaluation	(80)
**5**	Feedback integration	NLP-based feedback systems	Analyses student and faculty feedback at scale	Continuous curriculum refinement	Increased responsiveness to learner needs	(81)
**6**	Resource optimization	Predictive analytics	Forecasts resource needs (faculty time, modules, simulations)	Efficient allocation of teaching resources	Improved institutional efficiency	(5)
**7**	Continuous quality monitoring	AI-driven dashboards	Real-time tracking of curriculum effectiveness metrics	Ongoing curriculum evaluation and updating	Sustained quality improvement	(78)

### Data quality and trustworthy AI

The capability of AI in the creation of trustworthy classroom assessments and learning activities is reliant upon the overall quality, integrity, and representativeness of fundamental data (Table 4). Using data of poor quality due to missing, incomplete, inconsistent, biased (e.g., data sets) will produce predictions that will not be accurate, therefore resulting in poor decision making and ultimately achieving a negative impact on educational outcomes (i.e., grade level/or level of triumph) (32). Therefore, it is vital to ensure that data used within AI products is of superior quality to make AI effectively reliable. The establishment of new models or frameworks, such as the METRIC model, emphasizes various important elements such as bias detection, transparency, interpretability, and completeness (33). These components are essential to the development of a trustworthy AI product, which can thus be incorporated into medical education with a level of confidence. Additionally, using an explainable AI (XAI) methodology, educators will be provided with an understanding of how decisions were reached. Therefore, increasing their levels of trust and willingness to purchase. Many other issues exist outside data quality and AI development, which also need to be addressed to effectively utilize/implement AI-based systems, including data privacy, security, and ethical use of data (34). The design of applicable regulatory factors and strong management of sensitive clinical/educational data are also extremely vital to the use of data-based systems. Overall, the design of trustworthy AI systems will require a proper mix of technological robustness and appropriate ethics (35).

**Table 4:** Key Principles for trustworthy AI in medical education.

Sr. no.	Principle	Definition	Key considerations	Implications for medical education	References
**1**	Fairness	Ensuring AI systems do not produce biased or discriminatory outcomes	Bias detection, representative datasets, equitable model training	Promotes equal learning opportunities and unbiased assessment outcomes	(82)
**2**	Accountability	Clear assignment of responsibility for AI-driven decisions	Human oversight, governance frameworks, and audit mechanisms	Ensures educators and institutions remain responsible for AI use	(50)
**3**	Transparency	Openness in how AI systems function and make decisions	Explainable AI (XAI), model interpretability, and clear documentation	Builds trust among educators and learners, improves acceptance	(83)
**4**	Data quality	Use of accurate, complete, and reliable datasets	Data validation, standardization, consistency checks	Enhances reliability of AI-driven insights and educational decisions	(84)
**5**	Privacy & security	Protection of sensitive learner and patient data	Data encryption, anonymization, and compliance with regulations	Safeguards confidentiality and maintains ethical standards	(85)
**6**	Explainability	Ability to understand and interpret AI-generated outputs	Model transparency tools, interpretable algorithms	Enables informed decision-making by educators	(83)
**7**	Robustness	Stability and reliability of AI systems under varying conditions	Error handling, model validation, resilience testing	Ensures consistent performance across diverse educational settings	(86)
**8**	Ethical compliance	Alignment with ethical standards and professional guidelines	Ethical review boards, consent protocols, and fairness policies	Supports responsible integration of AI in medical training	(86)

### Faculty readiness and change management

Several factors play an important role in the successful integration of AI and data-driven systems into medical education, including the readiness of faculty and the way that institutions manage change (36). For educators to become competent in using AI, they need to possess the core competencies of having AI literacy, understanding how to interpret data, and having the ability to incorporate digital tools into their teaching practice by participating in faculty development programs including structured training in AI tools, learning analytics, and digital pedagogy that will provide them with both technical and pedagogical skills necessary for the successful development of an AI-enabled learning environment (Table 5) (37).

**Table 5:** Faculty competencies and challenges in AI integration.

Sr. No.	Domain	Key elements	Implications for medical education	References
**1**	AI literacy	Understanding AI concepts, limitations, and applications	Enables informed use and critical evaluation of AI tools	(37)
**2**	Data interpretation	Ability to analyze learning analytics and dashboards	Supports evidence-based teaching and intervention strategies	(87)
**3**	Pedagogical adaptation	Integration of AI tools into teaching methods	Enhances personalized and competency-based learning	(88)
**4**	Technical skills	Use of AI platforms, simulation tools, and digital systems	Facilitates effective implementation of AI-enabled education	(89)
**5**	Resistance to adoption	Fear of complexity, lack of trust, workflow disruption	Requires training, awareness, and institutional support	(40)
**6**	Change management	Faculty training programs, leadership support, policy frameworks	Ensures smooth and sustainable digital transformation	(90)

In addition, there is considerable resistance to adopting AI and data-driven systems into teaching due to the perception of system complexity, lack of transparency, and disruptions to traditional teaching workflows. Additionally, there is variability in digital proficiency between faculty, presenting another challenge to implementing AI and data-driven systems into curricula (38). To overcome these barriers, structured training, ongoing institutional support, and clearly outlined policies must be established.

In addition, effective implementation of AI and data-driven systems requires redesigning faculty workflows such that the data sources and respective AI systems are incorporated into the curriculum in a manner that does not add to the workload of the faculty. Leadership at the institutional level is critical to providing an innovative and collaborative environment, and engaging faculty will be absolutely necessary to achieve sustainable and effective AI-driven transformation in medical education (39).

## Current applications (emerging trends)

### Digital twins and advanced simulation

**Figure 3:**
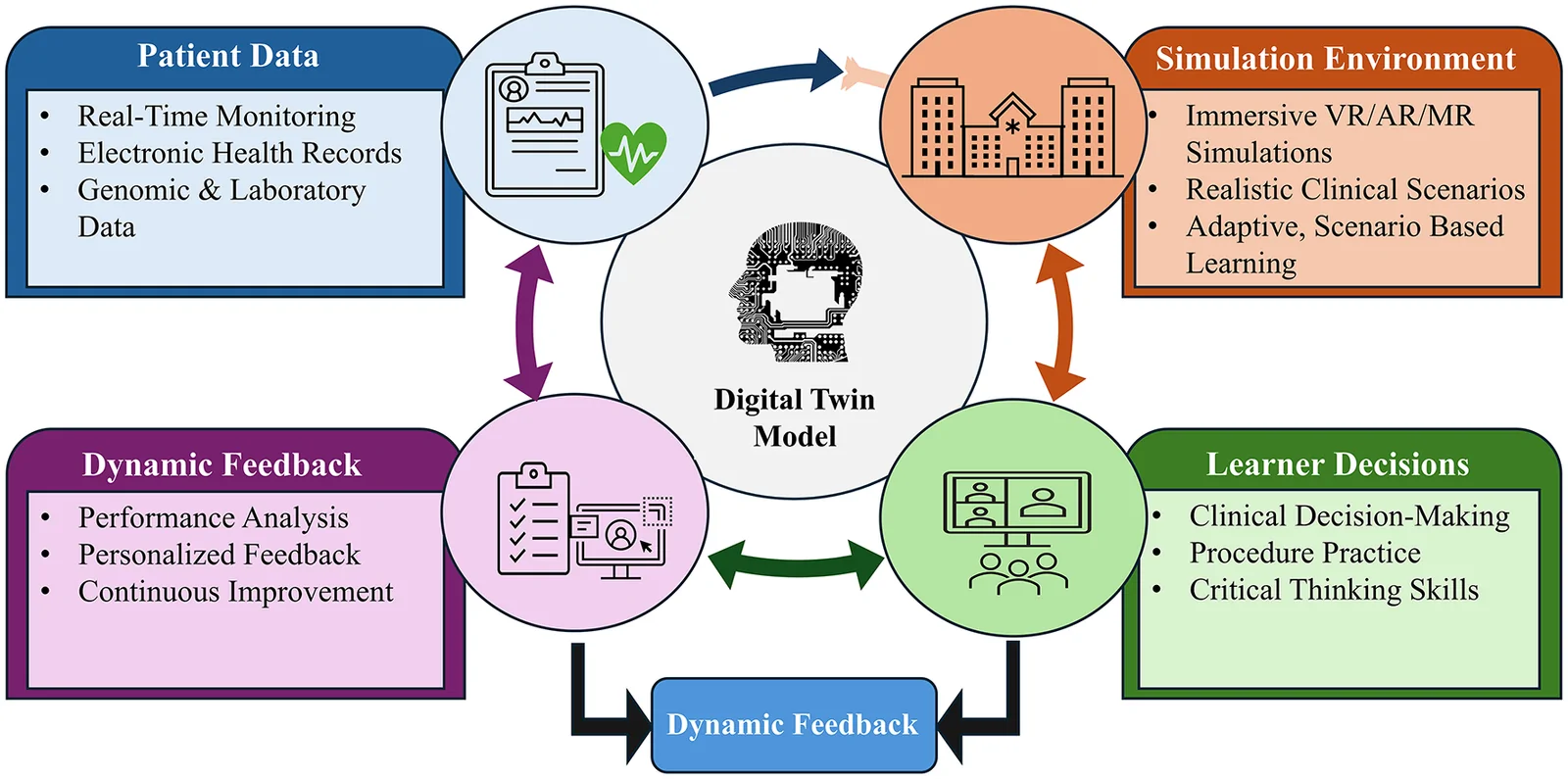
Conceptual framework of a digital twin–based clinical training system in medical education.

Digital twins and advanced simulation technologies are emerging in medical education as data-driven approaches that enable realistic and adaptive clinical training environments (39). These systems integrate clinical data and artificial intelligence (AI) to create realistic simulations of complex patient scenarios that allow learners to practice making clinical decisions in a controlled, safe setting with the ability to repeat their practice as well as standardize their training. In addition, the use of AI-enabled simulation platforms, such as virtual patients and immersive simulation environments, makes it possible for learners to engage in repeated practice and receive standardized training (20). The use of technologies such as virtual reality or natural language processing further enhances the fidelity or effectiveness of simulation-based education (See Figure 3).

### AI literacy and curriculum integration

With AI increasingly being integrated into healthcare systems, it is crucial to provide future healthcare providers with important competencies in AI. Understanding how to use AI in healthcare requires more than just technical understanding; it also requires a critical eye to appraise the importance of the ethical use of AI in healthcare settings. In contemporary literature, AI competencies are described as needing a longitudinal integration across all phases of medical education (40). At the beginning of medical education, learners are exposed to foundational elements of AI technology, such as machine learning, reading and interpreting data, and algorithmic thought. During the remainder of medical education, learners are exposed to the application of using AI for diagnostic and decision-support systems (and patient management). Furthermore, ethical considerations (such as data bias, data privacy issues, and accountability) are incorporated into AI training to help ensure that AI technologies are used responsibly (41). Interdisciplinary collaboration (i.e., working with peers in medicine, data science, engineering, and informatics) is encouraged so that all graduates will be both users of AI technologies and also informed decision-makers, capable of critically evaluating AI-generated outputs (Table 6) (42). In addition, integrating AI education into medical curricula requires alignment with existing competency-based frameworks and continuous evaluation to ensure its effectiveness and relevance in clinical practice (Table 7).

**Table 6:** Ethical challenges in AI-driven medical education and mitigation strategies.

Sr. no.	Ethical challenge	Underlying cause	Potential risk	Mitigation strategy	References
**1**	Algorithmic bias	Non-representative datasets	Unfair or unequal assessment outcomes	Use diverse data, perform bias audits	(91)
**2**	Lack of transparency	Black-box AI models	Reduced trust and interpretability	Apply explainable AI (XAI), clear documentation	(92)
**3**	Accountability issues	Unclear responsibility frameworks	Difficulty addressing errors	Define governance and oversight mechanisms	(93)
**4**	Data privacy concerns	Use of sensitive learner/patient data	Data breaches and misuse	Ensure anonymization, encryption, and compliance	(85)
**5**	Informed consent	Limited awareness of data usage	Ethical violations	Implement transparent consent processes	(94)
**6**	Over-reliance on AI	Excessive dependence on automation	Reduced critical thinking	Maintain human oversight	(95)
**7**	Inequity in Access	Unequal infrastructure availability	Digital divide in education	Promote equitable access and infrastructure	(96)

**Table 7:** Framework for AI Literacy integration in medical education.

S. no.	Level of training	Core competencies	Key learning components	Implementation strategies	Expected outcomes	References
**1**	Foundational (preclinical)	Basic AI concepts, data literacy, digital health awareness	Introduction to AI, machine learning basics, data interpretation	Lectures, e-learning modules, introductory workshops	Improved understanding of AI fundamentals and digital health tools	(97)
**2**	Intermediate (early clinical)	Application of AI in diagnostics and decision support	Clinical AI tools (e.g., imaging AI, CDS systems), data-driven decision-making	Case-based learning, simulation exercises, and guided tool usage	Ability to interpret and apply AI outputs in clinical scenarios	(98)
**3**	Advanced (clinical training)	Critical evaluation and integration of AI in patient care	Model limitations, bias detection, validation of AI outputs	Clinical rotations with AI tools, interdisciplinary training	Enhanced clinical reasoning and safe AI-assisted decision-making	(99)
**4**	Ethical & regulatory training	Understanding ethical, legal, and governance aspects of AI	Data privacy, bias, accountability, and regulatory frameworks	Seminars, policy discussions, ethics modules	Responsible and ethical use of AI in healthcare practice	(86)
**5**	Research & innovation level	AI development, validation, and translational research	Algorithm design basics, data handling, and research methodologies	Research projects, collaborations with data scientists	Ability to contribute to AI innovation and evidence-based practice	(100)
6	Continuing medical education (CME)	Lifelong learning and adaptation to emerging AI technologies	Updates on AI advancements, new tools, and clinical integration strategies	Workshops, online certification courses, and professional training programs	Sustained competency and adaptability in evolving digital healthcare	(57, 101)

### Continuous quality assurance systems

Quality assurance systems that are continuously implemented in medical training are increasingly being developed as data-based methods. A composite assessment model will be developed through learning analytics, AI-based assessments, and institutional data (43). This continuous quality assurance paradigm is increasingly applied to the entire spectrum of physician career development. A key trend is the focus on “career transition pathways,” such as facilitating the movement of specialists into general practice to meet primary care demands within tiered healthcare systems (44). Investigating the effectiveness of such transition training, through layered evaluation of knowledge acquisition, skill application, and long-term impact on healthcare delivery, represents a vital area of data-driven research. The insights gained help optimize training models, ensure the reliable conversion of clinical expertise across domains, and support lifelong learning portfolios (45). With real-time data analytics being collected on learner performance, learners will receive constructive and timely feedback, and adaptive interventions can be implemented to ensure that each student's needs are met. These systems improve transparency and accountability and facilitate personalized career paths within the field of medicine (46). Current examples of implementation in this area include analytical dashboards that allow schools to monitor students' progress, as well as competency-based evaluation systems (47). However, the extent to which any one of these methods has been adopted varies greatly by institution and is influenced by both the financial and digital readiness of the institutions.

## Ethical, legal, and implementation challenges

### Ethical concerns

Integrating AI into medical education presents ethical problems that require careful consideration to provide a responsible and equitable method of using AI. There are concerns around algorithmic bias due to imbalanced or unrepresentative training datasets, which cause an inequitable experience for different races when learning or testing (Table 6). Furthermore, equity concerns extend beyond algorithmic bias to encompass systemic disparities in educational delivery. Even in data-driven, non-AI training programs, significant heterogeneity in outcomes can arise from variations in training resources, faculty expertise, and curriculum implementation across different institutions. For instance, research on transition training programs has highlighted inconsistent competency attainment among graduates from different training sites, which risks perpetuating uneven quality of care in the primary care network This underscores the ethical imperative to ensure fairness and standardization as foundational principles in any data-informed educational reform, serving as a critical guardrail for subsequent AI integration. Additionally, many AI models lack transparency (especially black box models), preventing those who are using them from being able to justify their use of AI, which negatively impacts the trust and acceptance of those using AI (48, 49). Accountability for decision-making made through an AI system creates further complexity around ethical governance, as it is often unclear whether responsibility and potential liability lie with the developers, institutions, or educators using AI-assisted assessment systems (50). The use of AI in high-stakes assessments causes the problems mentioned above to be even more significant when considering that errors and biases associated with using AI could negatively impact the ability of students to move on to the next level academically or professionally. Therefore, fairness, inclusivity, and explainability must be achieved to ethically adopt AI within education (51). It is necessary to have ethical guidelines, ongoing evaluations, and a human oversight mechanism implemented within AI systems to reduce these risks and to ensure patient safety during AI-assisted clinical training and assessment.

### Data privacy and security

The implementation of AI technology for educational purposes will involve the collection, handling, and management of a large amount of sensitive data. Most often this includes information about students and their grades; however, in some situations, there may also be a need to collect and/or manage data from patients affected by a student's actions (52). Given the sensitivity of these data, issues of confidentiality, privacy, security, and overall ethical behavior arise. To ensure a sufficient level of ethical behavior when using AI tools, there must be strict data governance and management protocols (e.g., de-identification, encryption, and secure storage), which directly relate to the level of compliance with applicable national and international data protection laws (e.g., GDPR similar requirements) (53). Therefore, since obtaining informed consent from individuals and ensuring the transparency in the purpose of the data being used for other than originally intended purposes are considered critical aspects of ethical practice, the privacy concerns associated with these data will therefore present significant obstacles to the successful implementation of AI technologies in the field of medical education (54).

### Technical and institutional barriers

**Figure 4:**
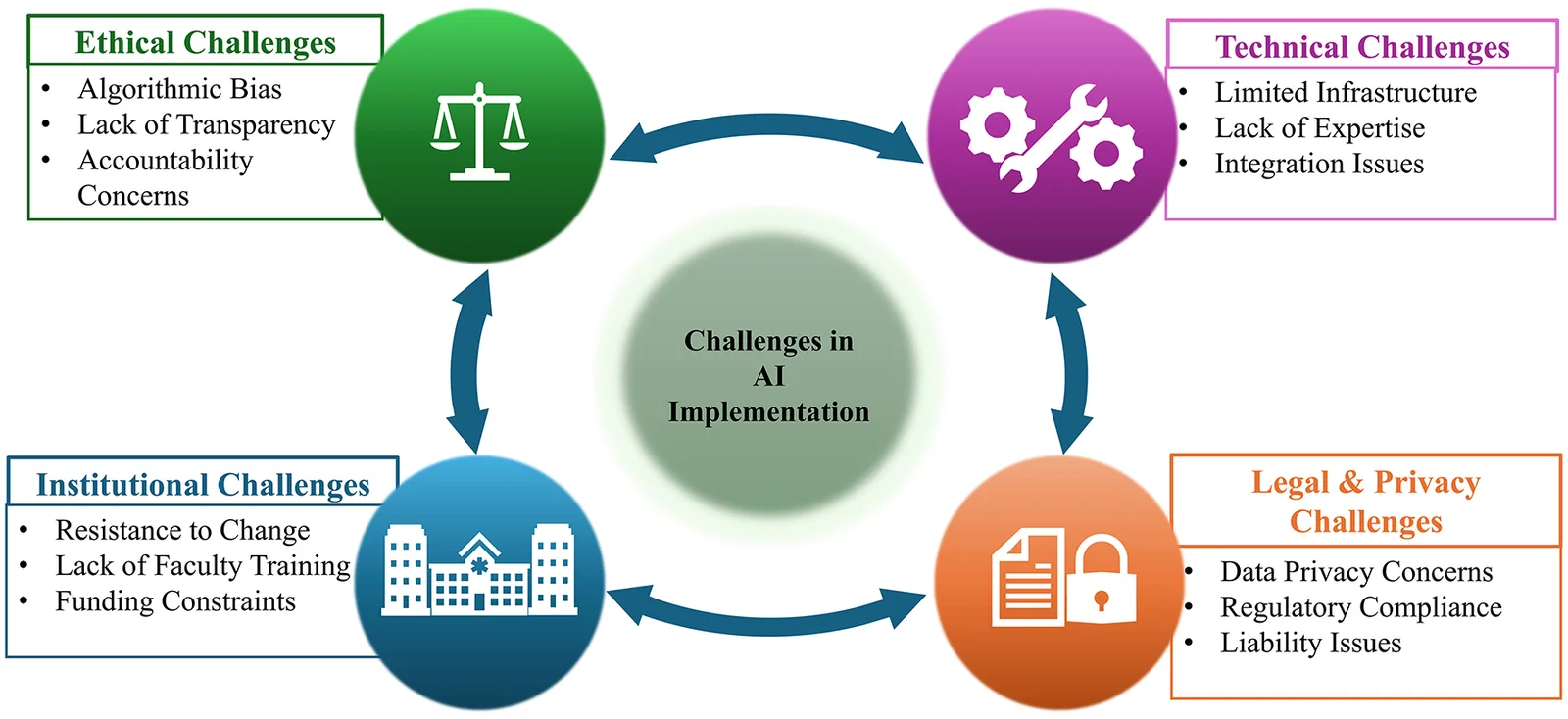
Barriers to the implementation of artificial intelligence in medical education categorized into four interconnected domains: ethical, technical, institutional, and legal/privacy challenges.

Apart from the legal and ethical issues, there are legal issues as well as there are many technical and systemic barriers to the effective use of AI in teaching within the clinical setting (Figure 4). The digital infrastructure is generally limited in many areas (particularly in developing nations), making it difficult to access AI services and platforms (55). In addition, the lack of adequately trained faculty and staff makes it difficult for institutions to implement and use AI systems effectively. The institutional resistance to making changes, combined with an inadequate policy framework, complicates the adoption. AI cannot be effectively integrated into existing curricula without significant alignment with approved educational objectives, accreditation standards, and custom instructional methodologies. In addition, there is still very little empirical data regarding the long-term impact and/or cost-benefit ratio of AI Educational Interventions (56).

### Future directions

As AI continues to advance and develop in ways that are better suited to providing comprehensive solutions for integrating education with work through technology, clinical education will continue to transform by being designed into more integrated and adaptive educational frameworks (57). In addition, many of these ongoing changes will likely create innovative future developments of digital ecosystems that will be developed to create interconnected systems of assessment, analytics, and institutional data in order to produce evidence-based and responsive decisions about education (58). Automation will most likely serve to support academic governance, specifically through creating more efficient systems for continuous benchmarking on program quality, accreditation, etc., via the use of continuous quality metrics (59). Furthermore, there is expected to be an emerging model for competency assessment that will utilize path-dependent mechanisms such as digital portfolios or credentialing systems, and will provide the foundation for supporting long-term professional development beyond traditional forms of education and credentialing (60). The use of advanced technology, such as simulations with greater accuracy due to its ability to create an individualized experience for each person, can also support more effective experiential learning through the creation of a more dynamic environment for training (4). As a result, the incorporation of artificial intelligence skills within medical education requires ongoing evaluation for effective use based on the new technologies and advanced clinical practices being developed. The future should focus on creating consistent assessment methods and testing the effectiveness (or success) of new educational tools based on AI. It is important to develop systems to evaluate all three factors of scalability, ethical governance, non-punitive, and institutional preparedness to fully realize the responsible and sustainable adoption of these innovations.

## Discussion

With the rapid growth and evolution of AI and data-centered technologies, there has been a major transformation in clinical teaching and medical education. Specifically, these technological advances have caused a shift in clinical education. This is referred to as a Transformational Change of Traditional Clinical Teaching (20). This article outlines how new AI-based teaching and learning systems are changing the way we teach, improving simulation-based training, objectively measuring clinical competence through data, and facilitating the development of interprofessional teams (61). Additionally, data-based systems used for quality assurance (learning analytics and predictive analytics) provide ongoing assessment of student performance and the effectiveness of curricula, providing evidence-based strategies for education, enhancing transparency, and enabling timely intervention for at-risk learners (27). The principles of data-driven quality assurance are also proving essential in addressing macro-level healthcare workforce challenges. As evidenced by research into national physician transition training programs, applying structured evaluation frameworks (e.g., Kirkpatrick model) and analytics to understand the determinants of training success is a critical step (44, 45). This work not only aims to improve specific training outcomes but also generates the robust, localized datasets necessary for future AI applications, such as predicting which clinicians are best suited for career transitions or personalizing their retraining pathways. Thus, foundational data analytics and advanced AI systems form a continuum in the digital transformation of medical education (62). While these advancements are occurring, there remain numerous barriers to the full implementation of these technologies, including, but not limited to, ethical concerns (i.e., algorithmic bias), lack of transparency in many AI systems, and privacy issues related to the use of student data (35). Other barriers include technological limitations (access), the lack of training for faculty, inadequate infrastructure for implementation, etc. AI implementation in medical education will also be impacted by resource disparities among healthcare settings. Tertiary hospitals and academic institutions typically have more access to digital resources and IT support than community health centers and primary care settings, thus creating a “digital divide” regarding the ability of these entities to implement AI-enhanced educational resources (63). AI evaluation models for healthcare professions should also address the differences between generalist and specialist training. Generalist training entails a broad base of knowledge in many clinical areas whereas specialized training has an in-depth knowledge base of the area of specialization. The lack of digital resources and support will inhibit AI's impact on medical education for both generalists and specialists. Therefore, AI-based evaluation models must be customized for each training environment to enhance the fairness and value of evaluations (64). After the analysis of all the selected studies, it can be concluded that the available evidence is heterogeneous. This heterogeneity arises from variations in study design, sample size, outcome measures, and the assessment of the effectiveness of different AI methods and tools over time. Therefore, the reported effectiveness of AI-based educational interventions should be interpreted with caution. Thus, it is necessary to develop standardized evaluation frameworks alongside conducting multicenter longitudinal studies in order to ensure the validity of the findings of studies dedicated to AI-based medical education (65). This review used two widely recognized research databases, PubMed and Scopus, to examine literature pertaining to the topic of AI and data-driven quality control as used in medical education. These databases ensured that a solid basis was established for the analysis of information about the subject in question. In order to make sure that future reviews build on this work, it would be advisable to include other data sources, such as Embase, ERIC, and the Cochrane Library, to further broaden the evidence base. In addition, future studies should concentrate on the modalities for the application of AI in different educational environments along with the establishment of reliable ethical governance mechanisms for the responsible, fair, and open use of AI in medical education. Although AI demonstrates considerable promise for improving medical education, the current evidence remains heterogeneous and is largely derived from observational and single-institution studies. Therefore, the reported benefits should be interpreted cautiously until supported by robust multicentre longitudinal investigations.

## Conclusion

Artificial intelligence, along with data-driven quality assurance systems, is changing how we train our Clinical and Medical Education Workforce by improving personalized learning, simulation-based training, and objective assessment of learners. Additionally, these technologies allow for ongoing performance monitoring, as well as optimization of the curriculum. The ethical implications and barriers to implementation must continue to be a concern. Future research on their potential to provide for long-term validation, scalability, and a standardized framework to allow for their responsible use will be necessary.
