# Supplementary material for: The digital transformation of clinical teaching: a review of AI and data-driven quality assurance systems in medical education
Source: Front Public Health. 2026 Aug 17;14:1915158. doi: 10.3389/fpubh.2026.1915158 (PMC13527599; doi:10.3389/fpubh.2026.1915158)
Supplement: Supplementary file 1 [file Data_Sheet_1.pdf]

Supplementary material S1. Detailed Literature Search Strategy and Study Selection Workflow

PubMed

Database: PubMed

Search date: 4 January 2026

Search query: "artificial intelligence" OR "machine learning" and "medical education" OR "clinical education" and "simulation" OR "clinical teaching" OR "assessment"

Filters applied

- Publication years: 2015–2026
- English language
- Article types:
    1. Journal Article
    2. Review
    3. Systematic Review
    4. Scoping Review
    5. Meta-analysis
    6. Evaluation Study

Scopus

Database: Scopus

Search date: 04 January 2026

Search query: "artificial intelligence" OR "machine learning" and "medical education" OR "clinical education" and "simulation" OR "clinical teaching" OR "assessment"

Filters applied:

- English
- 2015–2026
- Article
- Review

Reference Management and Study Selection

The records retrieved from PubMed (NBIB format) and Scopus (RIS format) were exported and imported into Mendeley Desktop for reference management. After applying the predefined database-specific filters, 710 records remained for further processing. Duplicate records were identified using Mendeley's duplicate detection function and verified manually, resulting in 654 unique records for title and abstract screening. Two reviewers independently screened the titles and abstracts according to the predefined inclusion and exclusion criteria. Following screening, 108 full-text articles were assessed for eligibility, of which 68 studies met the inclusion criteria and were included in the qualitative narrative synthesis. The study selection process is summarized in the PRISMA flow diagram (Figure 1).
